# Position of macula lutea and presence of proliferative vitreoretinopathy affect vitreous cytokine expression in rhegmatogenous retinal detachment

**DOI:** 10.1371/journal.pone.0234525

**Published:** 2020-06-15

**Authors:** Anikó Balogh, Tibor Milibák, Viktória Szabó, Zoltán Zsolt Nagy, Miklós D. Resch

**Affiliations:** 1 Department of Ophthalmology, Semmelweis University, Budapest, Hungary; 2 Department of Ophthalmology, Uzsoki Hospital, Budapest, Hungary; University of Pecs, HUNGARY

## Abstract

Our purpose was to evaluate the concentrations of vitreous cytokines in patients with rhegmatogenous retinal detachment (RRD). We hypothesized that patients with macula on RRD have lower levels of cytokines compared to patients with macula off RRD and proliferative vitreoretinopathy (PVR). Vitreous fluids were collected during 23G pars plana vitrectomy from 58 eyes of 58 patients. Indication for vitrectomy included macula off and macula on RRD, PVR, and idiopathic epiretinal membrane (ERM). A multiplex chemiluminescent immunoassay was performed to measure the concentrations of 48 cytokines, chemokines, and growth factors. Levels of HGF, IL-6, IL-8, IL-16, IFN-gamma, MCP-1, and MIF were significantly higher in all groups of retinal detachment compared to ERM. Levels of CTACK, eotaxin, G-CSF, IP-10, MIG, SCF, SCGF-beta, SDF-1alpha were significantly higher in PVR compared to macula on RRD and ERM. Levels of IL-1ra, IL-5, IL-9, M-CSF, MIP-1alpha, and TRIAL were significantly higher in PVR compared to macula on RRD. Our results indicate that the position of macula lutea and the presence of PVR significantly influence vitreous cytokine expression. The detected proteins may serve as biomarkers to estimate the possibility of PVR formation and may help to invent personalized therapeutic strategies to slow down or prevent PVR.

## Introduction

In rhegmatogenous retinal detachment (RRD), liquified vitreous enters under the neurosensory retina through a retinal break, which leads to the separation of the photoreceptor outer segment from the underlying retinal pigment epithelium (RPE). When vitreous reaches the retinal cells, the affected cells start to secrete factors involved in the destruction and survival of retinal structures. [1] RPE cells can migrate into the vitreous cavity and, as a pluripotent cell, can transform into macrophages. [2]

Kaufman at al. were among the first to report that macular involvement and duration of retinal detachment were major parameters for postoperative visual acuity. [3] Despite anatomically successful retinal detachment surgery resulting in reattached retina visual acuity remains impaired in almost 40% of cases, especially when the macula was detached or proliferative vitreoretinopathy (PVR) developed after surgery. [4]

Retinal detachment can cause vision loss if untreated, and even despite proper surgical intervention, a potentially sight-threatening condition may develop in some cases. Even with a high success rate of primary vitrectomy for RRD [5] one of the most difficult challenges for vitreoretinal surgeons is the management of PVR. Therefore, the pathophysiology of PVR is under research, including cytokines, chemokines, and other inflammatory factors. [6] Many groups try to explore the possible non-surgical treatment of PVR, e.g. Pennock et al. proposed that ranibizumab might be potential prophylaxis for PVR. They discovered that ranibizumab reduced the bioactivity of vitreous of patients and experimental animals with PVR, and protected rabbits from developing the disease. [7] Other groups studied further agents that may be effective in the treatment of PVR. Kunikata et al. investigated the role of intravitreal injection of triamcinolone acetonide (IVTA) in preventing photoreceptor apoptosis in eyes with RRD. They discovered that IVTA suppressed elevated levels of aqueous humour MCP-1, MIP-1β, and IP-10 in eyes with RRD. [8] Asaria et al. found that adjuvant 5-fluorouracil and low molecular weight heparin significantly reduce the incidence of postoperative PVR. [9] Sadaka et al. evaluated intravitreal methotrexate infusion during pars plana vitrectomy for RRD with a high risk of PVR. They concluded that eyes at high risk for PVR had a low incidence of PVR formation following intravitreal methotrexate infusion. [10] Kawahara et al. suggested that statins could be potent inhibitors of cicatricial contraction in proliferative vitreoretinal diseases. They found that intravitreal injection of simvastatin dose-dependently prevented the progression of diseased states in an in vivo model of PVR. [11] Some groups established animal models of PVR that allows extensive functional studies and drug testing. Márkus et al. studied the role of transglutaminase 2 in a knockout mouse model of PVR, and they found that the lack of transglutaminase 2 did not prevent the formation of PVR. [12] Despite these findings, there is no available cure or prophylaxis for PVR as of yet, apart from the surgical approach. [13]

The purpose of this study was to explore the immunological components that are responsible for the proliferative alterations in RRD and to compare the differences in the levels of vitreous cytokines, chemokines, and growth factors of eyes with macula on, macula off RRD and PVR. The detected proteins may serve as biomarkers to predict the possibility of PVR formation and may help to invent personalized therapeutic strategies to slow down or prevent PVR.

## Materials and methods

### Subjects

The present study was approved by the Hungarian Medical Research Council Committee of Science and Research Ethics (Approval No. 15028-2/2017/EKU) and performed in accordance with the tenets of the Declaration of Helsinki. All participants gave written informed consent to the study.

Fifty-eight eyes of 58 patients, who underwent pars plana vitrectomy, at two vitreoretinal centres between January 2017 and June 2018, were studied prospectively. Indication for vitrectomy included macula off (n = 16) and macula on (n = 13) rhegmatogenous retinal detachment, rhegmatogenous retinal detachment with proliferative vitreoretinopathy (n = 13) and idiopathic epiretinal membrane (n = 16). Patients demographics and clinical data are summarized in **Table 1** and **Table 2.** Patients who had a history of previous vitreoretinal surgery, penetrating injury, uveitis, aphakia, age-related macular degeneration, diabetic retinopathy, and uncontrolled glaucoma were excluded.

**Table 1:** Demographic data of the patients in the groups.

	PVR	RRD off	RRD on	ERM
N	13	16	13	16
Male/Female	6/7	11/5	8/5	5/11
Age in years (mean±SD)	58.3± 16.3	63.9 ± 7.1	58.6 ± 10,3	68.6 ± 11.6

**Table 2:** Clinical data of patients.

	Symptom duration (day) mean ± SD	The extent of RD (quadrants)		Location of the tear (%)			PPV endotamponade (%)		
			superior	inferior	temporal	nasal	SF6	C3F8	SIO
PVR	21 ± 19.4	3.07 ± 0.95	45.4	36.4	0	18.2	15.4	53.8	30.8
RRD off	8.1 ± 7.4	2.28 ± 0.73	42.85	14.3	42.85	0	12.5	62.5	25
RRD on	5.8 ± 4.8	1.5 ± 0.46	69.2	0	23.1	7.7	7.7	84.6	7.7

PPV: pars plana vitrectomy; SIO: silicon oil; SF6, C3F8 gas tamponade

### Vitreous sample preparation

Undiluted vitreous samples were collected during a standard three-port 23G pars plana vitrectomy by two surgeons (MR and TM). During core vitrectomy, vitreous fluid (0.5 ml) was collected from the eyes before starting irrigation. The samples were stored in Eppendorf tubes, cooled in a freezer at -20°C for some hours, and then frozen at −80°C until the assay was performed.

### Cytokine analysis

Vitreous samples were analysed using a multiplex bead-based immunoassay, the Bio-Plex system (Bio-Rad Laboratories, Hercules, CA, USA). Human Cytokine Screening Panel, 48-Plex (Bio-Rad Laboratories) was used to detect the molecules. The vitreous fluid was diluted fourfold through the use of sample diluent provided by the Bio-Plex beads array kit (Bio-Rad Laboratories, Hercules, CA). Dilution was chosen according to the relevant previous papers an expected range of concentrations. [14] [8] Samples were prepared by first centrifuging the specimen at 10,000 ×g for 5 min after vortex agitation. A total volume of 50 μL from each sample was used for the assay. The kits were used according to the manufacturer’s instructions by an experienced technician using the Bio-Plex 100 array reader with Bio-Plex Manager (software version 6.1); Bio-Rad Laboratories, Hercules, CA, USA).

Forty-eight molecules were measured, including cutaneous T-cell attracting chemokine (CTACK), eotaxin, basic fibroblast growth factor (basic FGF), granulocyte colony-stimulating factor (G-CSF), granulocyte–macrophage colony-stimulating factor (GM-CSF), growth-related oncogene alpha (GRO-alpha), hepatocyte growth factor (HGF), interferon alpha2 and gamma (IFN-alpha2 and IFN-gamma), IL-1alpha (interleukin), IL-1beta, IL-1 receptor antagonist (IL-1ra), IL-2, IL-2 receptor alpha (IL-2Ralpha), IL-3, IL-4, IL-5, IL -6, IL -7, IL -8, IL -9, IL -10, IL -12/p40, IL-12/p70, IL-13, IL -15, IL -16, IL -17, IL -18, interferon gamma–induced protein 10 (IP-10), leukemia inhibitory factor (LIF), monocyte chemotactic protein 1 and 3 (MCP-1, MCP-3), macrophage colony-stimulating factor (M-CSF), macrophage migration inhibitory factor (MIF), monokine induced by interferon gamma (MIG), macrophage inflammatory protein 1 alpha and beta (MIP-1alpha, MIP-1beta), beta-nerve growth factor (beta-NGF), platelet-derived growth factor (PDGF-BB), regulated upon activation, normal T cell expressed and secreted (RANTES), stem cell factor (SCF), stromal cell–derived factor 1alpha (SDF-1alpha), stem cell growth factor beta (SCGF-beta), tumor necrosis factor alpha and beta (TNF-alpha, TNF-beta), tumor necrosis factor–related apoptosisinducing ligand (TRIAL) and vascular endothelial growth factor (VEGF). The measurements of these cytokines were performed by using 96-well assay plates and reagent kits according to the procedure provided by the manufacturer.

### Statistical analysis

After calculating the cytokine concentrations in the specimens, a Kruskal–Wallis analysis of variance and Dunn’s multiple comparison test was performed, and P-values were calculated via dedicated statistical software (GraphPad Prism, La Jolla, CA). *P*-values <0.05 were set to indicate statistically significant results.

## Results

### Clinical data

Fifty-eight eyes of 58 patients were included in this study. Four groups of patients were formed as follows: a control group consisting of patients without RRD who underwent vitrectomy for the management of ERM, patients with macula off RRD with PVR-C [15], patients with macula off and patients with macula on RRD without PVR. **Table 1** shows the patient’s demographic data in the groups. The differences in age between the groups were not statistically significant.

### Vitreous cytokine, chemokine, growth factor patterns

A total of 48 cytokines, chemokines, and growth factors were analyzed in the vitreous samples and compared between the four groups. An assay could be performed on all samples; **Table 3** lists the P values, median, and interquartile range (IQR) of concentrations of all individual cytokines in the four patient groups. A Kruskal-Wallis test and Dunn’s multiple comparison test selected 24 out of 48 cytokines, which reached the level of significance in concentration.

**Table 3:** Median concentrations (pg/ml) and interquartile range of cytokines, chemokines and growth factors in the vitreous of eyes with PVR, macula off and on RRD, and ERM.

	PVR Median (IQR) pg/mL	RRD off Median (IQR) pg/mL	RRD on Median (IQR) pg/mL	ERM Median (IQR) pg/mL	P Value
HGF	8135 (5695–11547)	7856 (5373–11877)	4730 (3611–7776)	134.4 (124.7–221.8)	<0.0001
IFN-gamma	67.8 (50.06–128.7)	82.14 (54.48–129)	44.23 (37.54–81.51)	29.2 (23.96–32.81)	<0.0001
IL-6	112.3 (43.42–280.8)	40.87 (25.66–208.4)	34.38 (14.03–115.7)	9.77 (6.05–13.75)	<0.0001
IL-8	120.5 (56.42–197.4)	81.52 (39.48–103.7)	34.69 (29.23–80.4)	28.23 (18.12–45.54)	0.0003
IL-16	54.39 (31.24–114.4)	37.46 (22.47–75.03)	48.38 (15.43–75.25)	16.71 (13.29–21.93)	<0.0001
MCP-1	1950 (1218–2687)	1996 (1066–2848)	1107 (798.7–1882)	379.4 (309.5–517.4)	<0.0001
MIF	4371 (3323–4701)	2967 (2036–4030)	2349 (1098–2870)	761.3 (606.9–1201)	<0.0001
CTACK	76.36 (56.23–103.4)	49.04 (27.73–75.62)	34.02 (22.13–51.68)	47.13 (35.19–66.34)	0.0012
Eotaxin	7.91 (6.025–10.25)	6.335 (4.683–8.035)	4.21 (3.465–5.46)	4.42 (3.073–6.1)	0.0006
G-CSF	129.9 (108.4–203.9)	130.7 (94.16–152.7)	76.3 (42.92–106.7)	87.4 (57.18–115.4)	0.0014
IP-10	958.1 (783.5–2208)	529.9 (304.1–1044)	354.8 (303.3–483.7)	249.1 (141.7–408)	<0.0001
MIG	205.3 (142.3–333.1)	104.5 (74.72–137.1)	53.91 (42.38–80.47)	58.07 (38.95–109.2)	<0.0001
SCF	87.46 (51.8–108)	62.55 (40.28–68.26)	31.6 (21.75–44.61)	48.21 (32.32–55.21)	0.0001
SCGF-beta	31569 (18211–58395)	16368 (6636–21651)	6625 (2120–11375)	10018 (6751–18778)	<0.0001
SDF-1alpha	242.9 (138.9–277.2)	94.31 (77.99–159.7)	63.69 (45.68–85.8)	70.11 (42.57–79.11)	0.0002
IL-1ra	97.77 (78.89–111.4)	76.01 (61.91–105.9)	60.72 (30.61–75.25)	73.08 (43.2–84.49)	0.0041
IL-5	73.72 (57.3–101.1)	61.1 (46.12–87.46)	32.84 (26.93–49.01)	47.73 (25.61–70.54)	0.0035
IL-9	22.32 (15.03–28.06)	16.33 (136.1–22.84)	11.92 (7.275–15.5)	14.25 (8.438–19.97)	0.0111
M-CSF	28.8 (17.1–32.35)	23.8 (17.1–32.56)	15 (10.8–22.12)	23.38 (16.89–30.89)	0.0114
MIP-1alpha	3.31 (2.31–3.965)	2.745 (2.018–3.648)	1.65 (1.07–2.43)	2.18 (1.34–2.99)	0.0046
TRIAL	14,76 (11,61–17,09)	13,19 (9,74–16,44)	9,475 (5,123–13,19)	11.6 (7.59–15.54)	0.0209
IL-1alpha	25.17 (16.21–34.94)	23.09 (10.79–32.83)	10.1 (4.775–13.48)	25.17 (11.44–39.15)	0.0202
IL-12(p40)	226 (207.2–370.9)	281.4 (207.2–335.5)	188.2 (101.6–260.5)	423.4 (226–492.1)	0.0174
IL-2Ralpha	36.52 (21.06–47.21)	11.09 (6.488–17.84)	6.49 (3.79–11.09)	19.28 (13.18–27.01)	<0.0001
Basic FGF	451.2 (395.4–598.7)	469 (242.1–574.4)	308.6 (148.6–418.3)	486.8 (253.8–577.2)	0.1397
GM-CSF	4.14 (3.1–6.51)	4.81 (2.37–6.27)	3.01 (2.328–5.205)	4.48 (2.74–7.38)	0.2559
GRO-alpha	163.7 (115.3–207.9)	147.7 (127.1–210.9)	124.7 (96.95–209.5)	134.4 (124.7–221.8)	0.5669
IFN-alpha2	22.78 (19.01–32.31)	23.67 (17.07–33.59)	11.03 (6.125–23.89)	20.94 (17.07–40.33)	0.1208
IL-1beta	4.06 (2.845–5.38)	3.92 (2.775–4.83)	2.03 (1.73–3.78)	4.34 (2.77–5.73)	0.1174
IL-2	10.8 (6.96–13.36)	12.5 (5.255–14.96)	4.83 (3.12–9.73)	10.37 (6.53–15.06)	0.0714
IL-3	1.06 (0.91–1.545)	1.27 (0.635–1.57)	0.71 (0.46–0.86)	1.19 (0.51–1.62)	0.0642
IL-4	1.74 (1.51–1.948)	1.695 (1.245–2.15)	0.83 (0.41–1.6)	1.88 (1.078–2.39)	0.0327*
IL-7	51.35 (36.04–70.14)	49.06 (28.81–69.6)	32.83 (22.03–47.58)	60.33 (32.35–83.88)	0.0556
IL-10	11.55 (8.088–17.43)	11.09 (6.488–16.01)	6.49 (3.79–9.01)	9.93 (5.355–19.57)	0.0536
IL-12(p70)	18.4 (12.07–29.98)	20.99 (7.9–27.24)	12.07 (5.993–15.43)	24.67 (14.87–40.88)	0.0562
IL-13	2.49 (1.93–2.895)	2.35 (1.215–2.963)	1.36 (1.07–2.21)	1.93 (1.07–2.76)	0.1156
IL-15	164.4 (134.5–232.3)	189 (150.6–226.6)	157.1 (126.7–173.2)	197.5 (134.3–245.8)	0.3047
IL-17	18.94 (14.95–25.93)	20.27 (12.47–26.27)	10.97 (6.99–17.28)	22.27 (11.3–33.93)	0.0662
IL-18	9.67 (6.54–15.3)	7.88 (5.7–12.15)	6.54 (4.08–9.68)	6.99 (3.973–7.88)	0.0495*
LIF	55.39 (36.43–70.42)	60.04 (34.03–91.99)	31.61 (7.538–61.18)	55.39 (24.25–64.67)	0.1480
MCP-3	4.6 (3.16–6.26)	3.89 (3.53–6.26)	1.97 (1.438–5.363)	4.6 (1.98–5.94)	0.2215
MIP-1beta	15.18 (6.68–21.16)	11.31 (2.715–20.04)	4.83 (3.845–17.65)	2.03 (0.03–4.07)	0.0704
beta-NGF	11.68 (7.175–14.33)	13.89 (8.09–17.8)	8.54 (3.67–13.01)	19.42 (9–22.78)	0.0504
PDGF-BB	75.19 (61.51–95.15)	74.14 (57.15–111.8)	74.17 (35.3–94.19)	72.03 (35.6–106.2)	0.9538
RANTES	22.13 (19.41–30.9)	23.2 (18–27.11)	18.28 (13–22.13)	24.26 (18.85–32.49)	0.0411*
TNF-alpha	24.44 (20.46–35.79)	27.08 (12.87–36.66)	14.67 (7.41–25.32)	25.32 (16.46–35.8)	0.1338
TNF-beta	10.87 (6.73–20.49)	12.32 (9.405–19.5)	5.52 (4.59–12.31)	7.93 (5.19–13.75)	0.1192
VEGF	239.2 (213.6–323.1)	263.9 (174.9–305.1)	193.1 (138.3–266.7)	265.3 (206.3–335.6)	0.1276

*: according to Dunn’s post hoc test there was no significant difference between the groups

P values in the table were calculated with the Kruskal-Wallis test

### Correlation of vitreous cytokine expression with the position of macula lutea and presence of PVR

**Fig 1:**
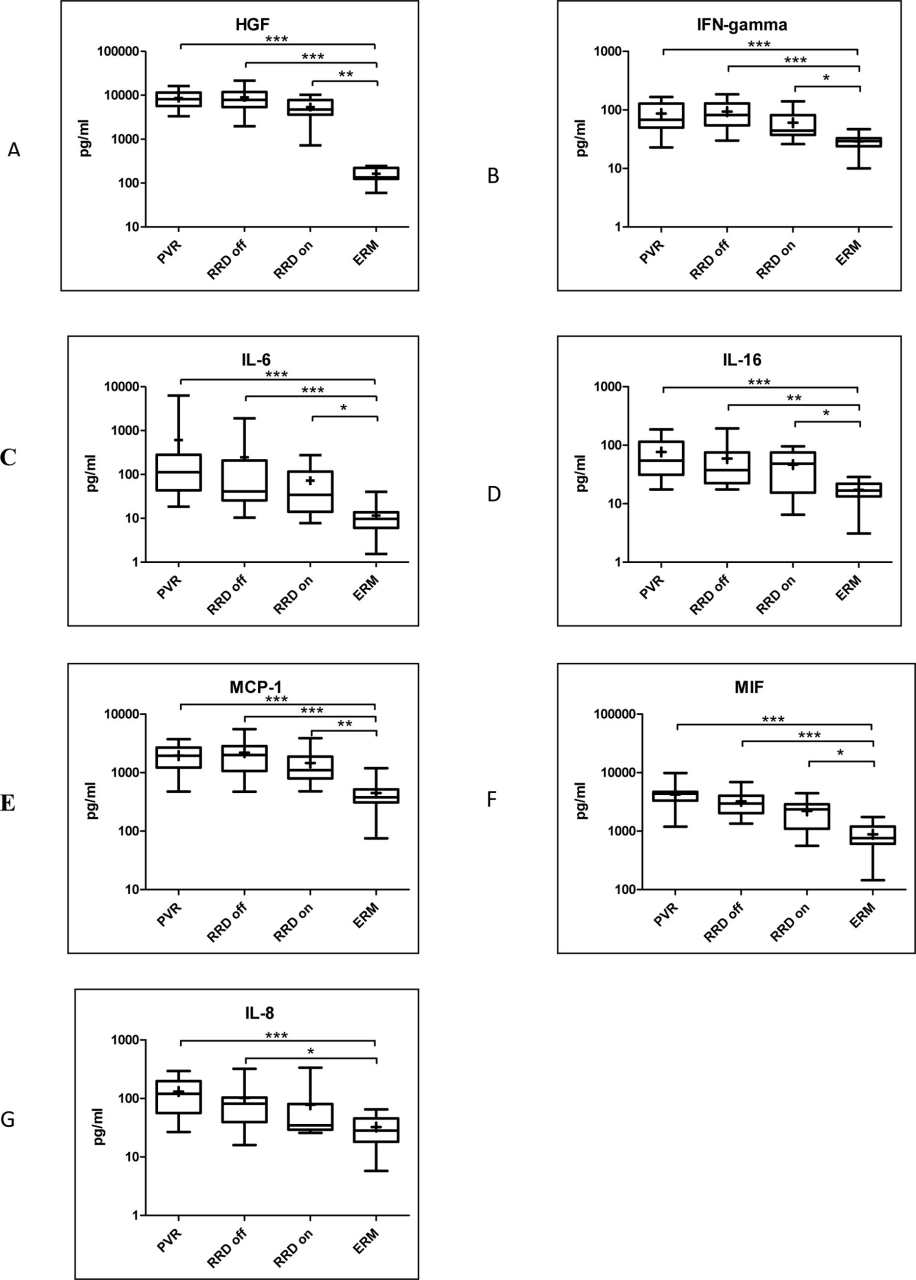
Upregulated molecules in PVR, macula off and on RRD compared to ERM. Median and mean (cross) concentrations of HGF, IFN-gamma, IL-6, -16, MCP-1, MIF, and IL-18 in eyes with PVR, macula off RRD, macula on RRD and ERM. Statistically significant differences between the groups are marked by an asterisk. * p<0.05; ** p<0.01; *** p<0.001.

**Fig 2:**
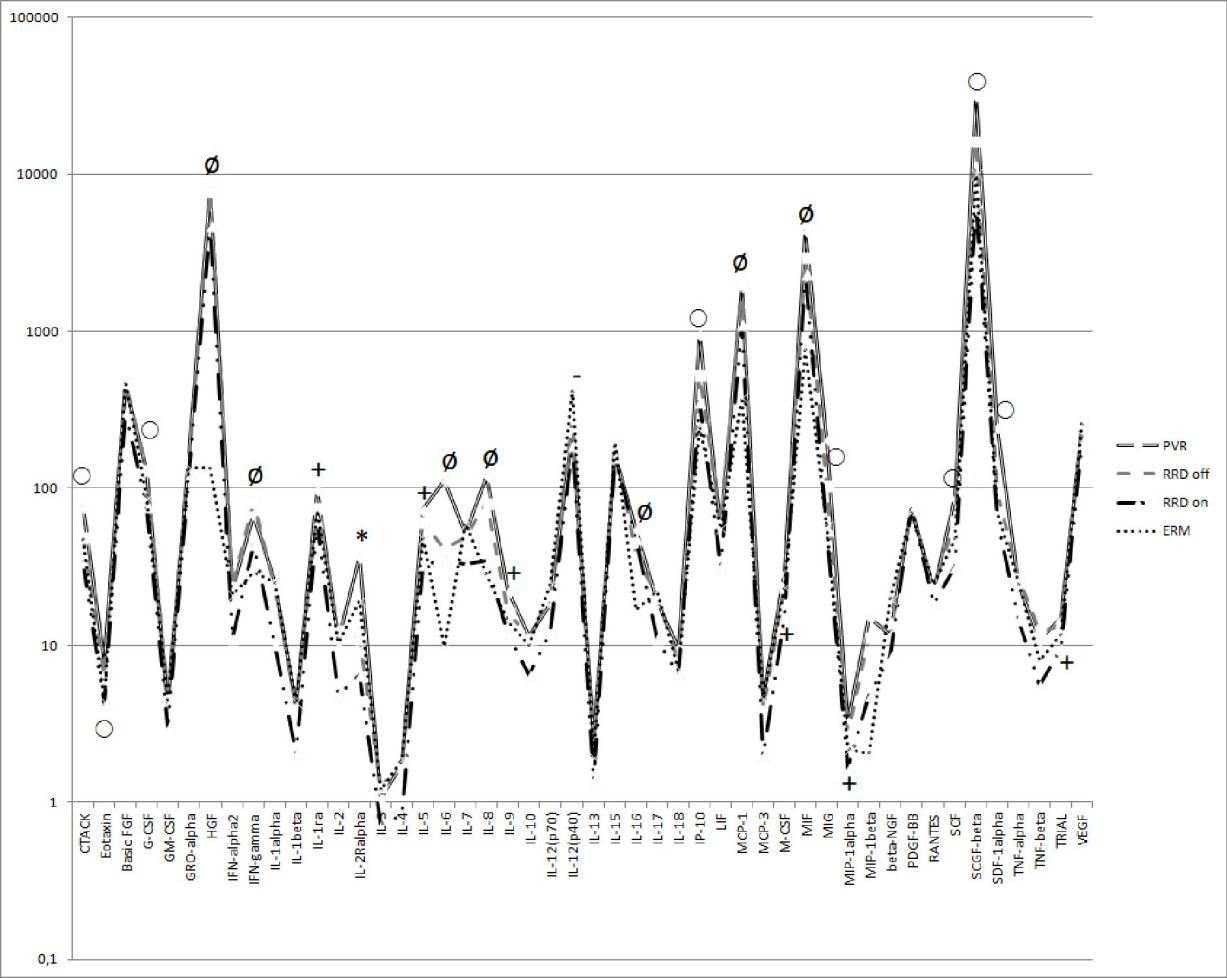
Cytokine levels in the vitreous fluid. Comparing cytokine levels in the vitreous of eyes of patients with proliferative vitreoretinopathy (PVR; n = 13), with macula off (RRD off; n = 16) and macula on (RRD on; n = 13) rhegmatogenous retinal detachment, and eyes with epiretinal membrane (ERM; n = 16). ø: Upregulated molecules in PVR, macula off and on RRD compared to ERM ○: Upregulated molecules in PVR compared to macula on RRD and ERM **+:** Upregulated molecules in PVR compared to macula on RRD **-:** Molecules which concentrations were significantly lower in macula on RRD compared to ERM *: IL-2 Ralpha upregulated in PVR compared to macula off and on RRD, and the concentration is significantly lower in macula on RRD compared to ERM.

Six molecules were upregulated in the case of all retinal detachment groups (PVR, macula off, and macula on RRD) compared to the control group. Levels of HGF (p<0.0001), IFN-gamma (p<0.0001), IL-6 (p<0.0001), IL-16 (p<0.0001), MIF (p<0.0001), MCP-1 (p<0.0001) were significantly higher in all groups of retinal detachment compared to the group of ERM. The concentration of IL-8 (p = 0.0003) was significantly higher in PVR and macula off RRD compared to the control group, but we could not find upregulation in macula on RRD **(Fig 1)**. The concentrations of three molecules out of six were higher than 1 ng/ml in all retinal detachment groups (median concentrations in PVR: HGF = 8.135 ng/mL, MCP-1 = 1.950 ng/mL, MIF = 4.371 ng/mL). **(Table 3; Fig 2)**

**Fig 3:**
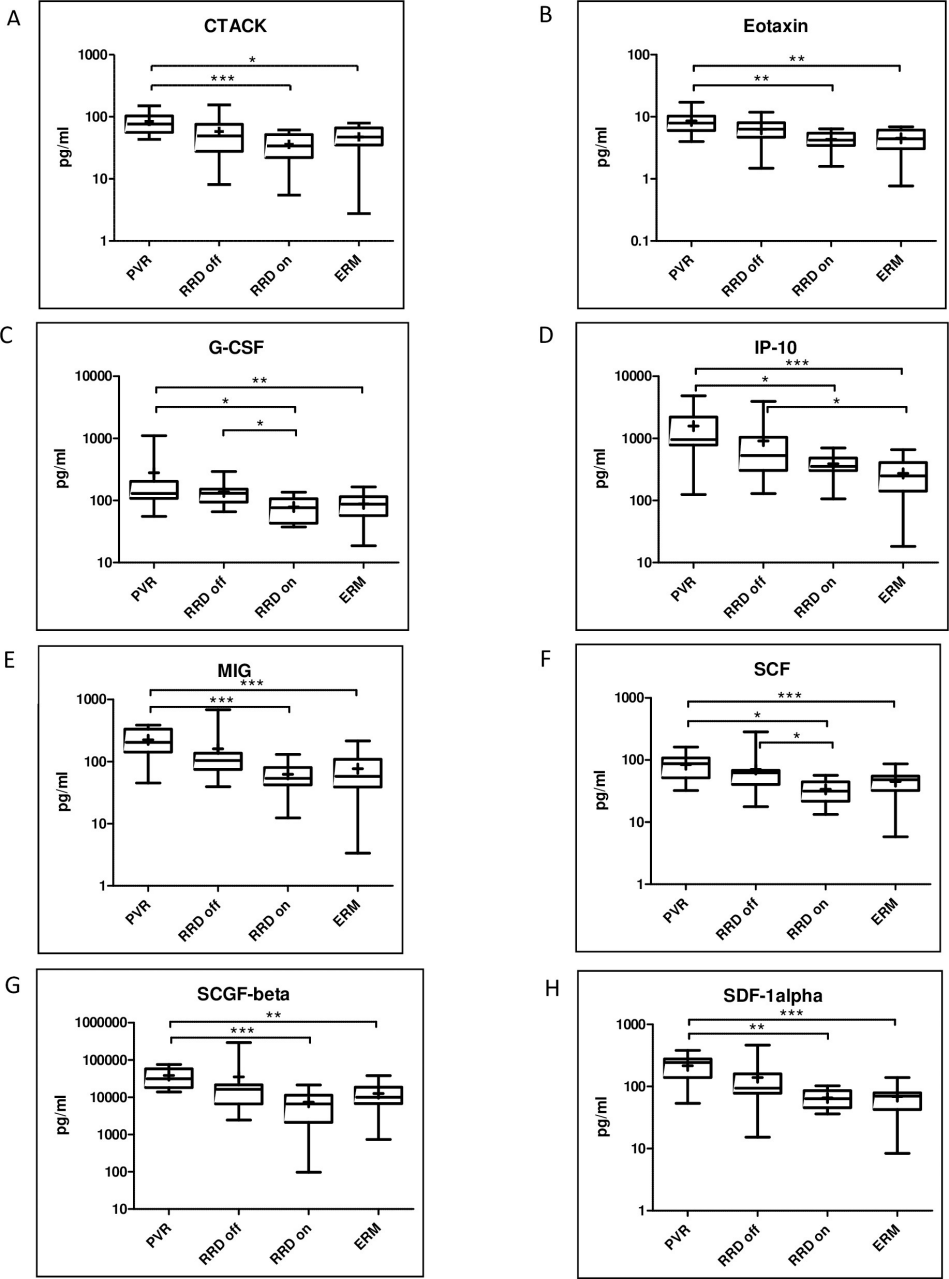
Upregulated molecules in PVR compared to macula on RRD and ERM. Median and mean (cross) concentrations of CTACK, eotaxin, G-CSF, IP-10, MIG, SCF, SCGF-beta, SDF-1alpha in eyes with PVR, macula off RRD, macula on RRD and ERM. Statistically significant differences between the groups are marked by an asterisk. * p<0.05; ** p<0.01; *** p<0.001.

There were eight significantly upregulated molecules in PVR compared to macula on RRD and ERM: CTACK (p = 0.0012), eotaxin (p = 0.0006), G-CSF (p = 0.0014), IP-10 (p<0.0001), MIG (p<0.0001), SCF (p = 0.0001), SCGF-beta (p<0.0001), SDF-1alpha (p = 0.0002) **(Fig 3)**. Levels of G-CSF and SCF were additionally upregulated in macula off RRD compared to macula on RRD **(Fig 3C and 3F)**. The concentration of IP-10 was significantly higher in macula off RRD compared to ERM as well **(Fig 3D)**. SCGF-beta exhibited the highest expression levels in PVR group (median concentration = 31569 pg/mL). Levels of four out of eight molecules were higher than 100 pg/mL (median concentration in PVR: G-CSF = 129.9 pg/mL, IP-10 = 958.1 pg/mL, MIG = 205.3 pg/mL, SDF-1alpha = 242.9 pg/mL). **(Table 3; Fig 2)**

**Fig 4:**
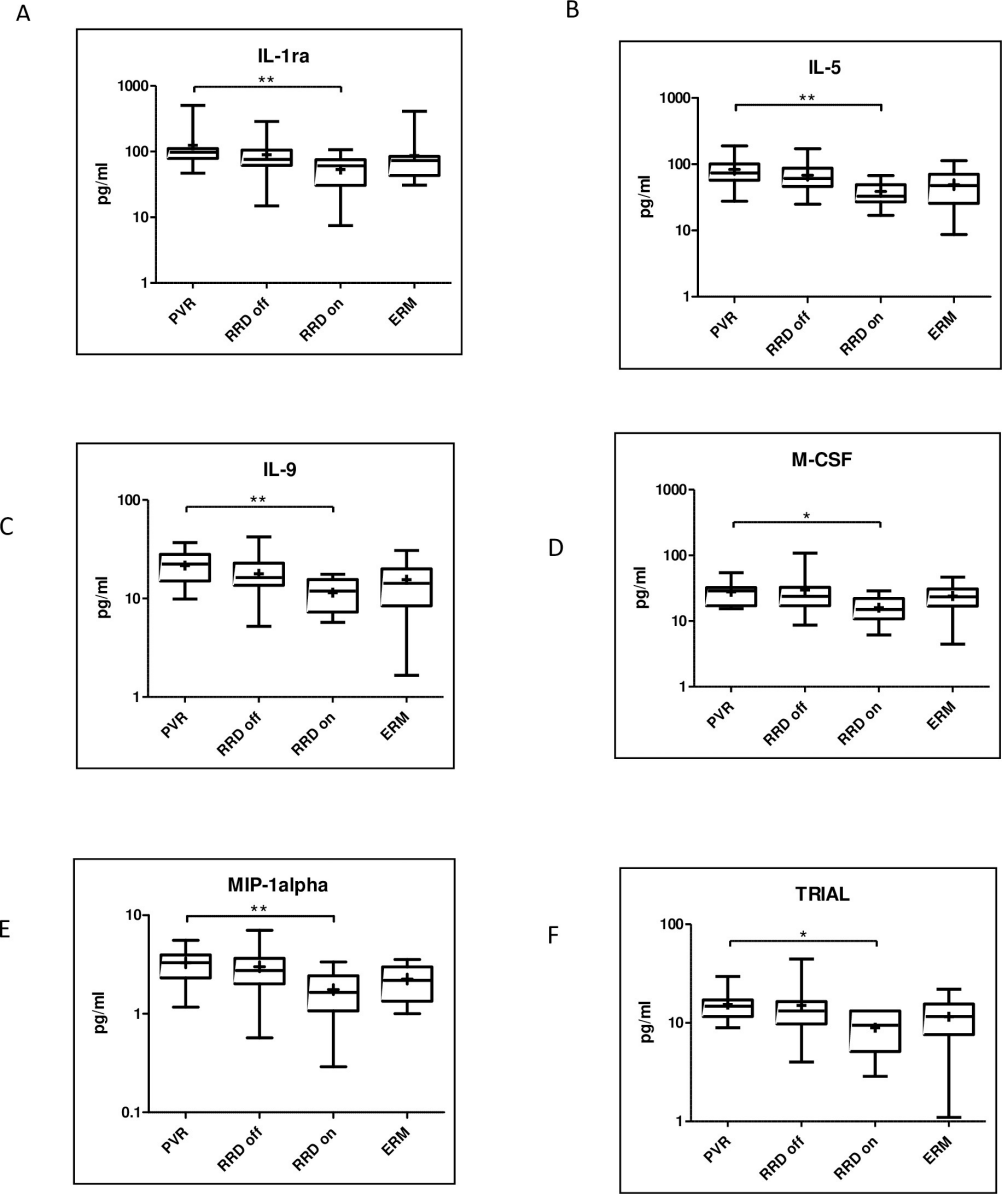
Upregulated molecules in PVR compared to macula on RRD. Median and mean (cross) concentrations of IL-1ra, -5, -9, M-CSF, MIP-1alpha, TRIAL in eyes with PVR, macula off RRD, macula on RRD and ERM. Statistically significant differences between the groups are marked by an asterisk. * p<0.05; ** p<0.01.

Concentration of six molecules were significantly higher in PVR compared to macula on RRD: IL-1ra (p = 0.0041), IL-5 (p = 0.0035), IL-9 (p = 0.0111), M-CSF (p = 0.0114), MIP-1alpha (p = 0.0046), TRIAL (p = 0.0209) **(Fig 4)**.

**Fig 5:**
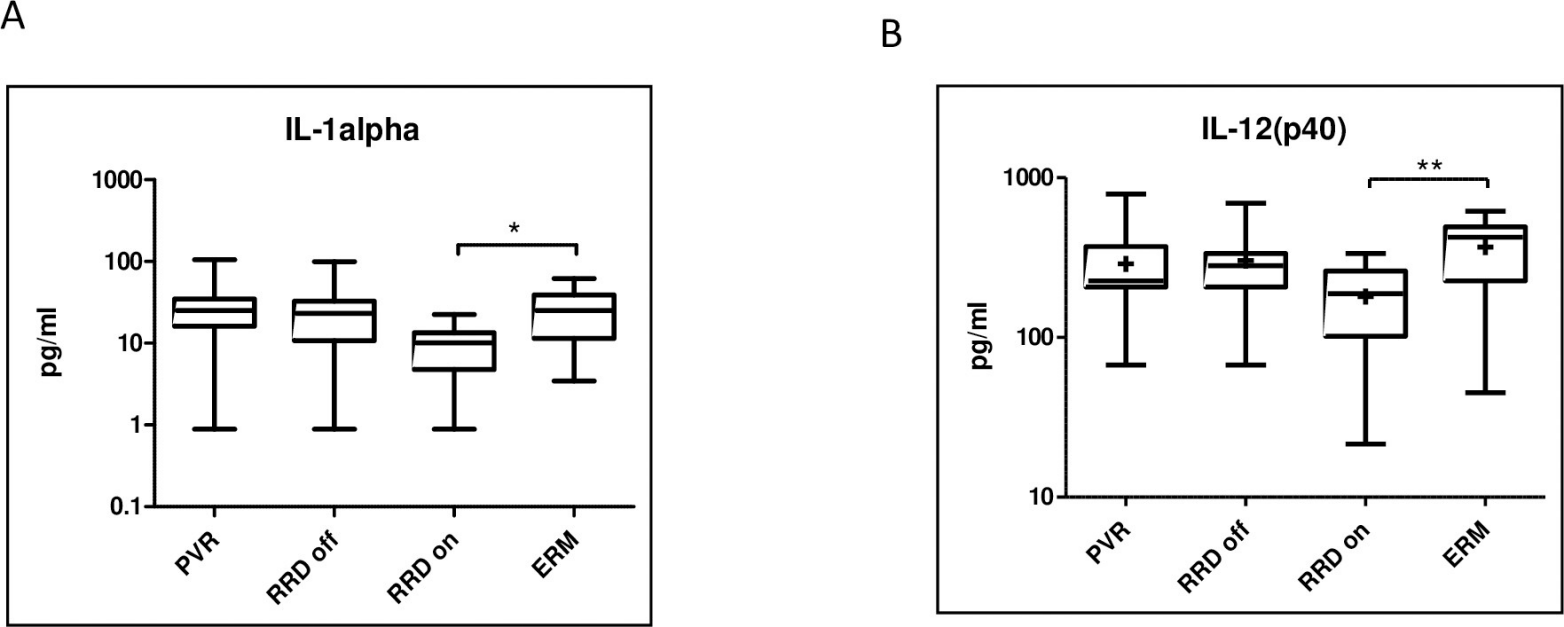
Molecules which concentrations were significantly lower in macula on RRD compared to ERM. Median and mean (cross) concentrations of IL-1alpha, IL-12(p40) in eyes with PVR, macula off RRD, macula on RRD, and ERM. Statistically significant differences between the groups are marked by an asterisk. * p<0.05; ** p<0.01.

**Fig 6:**
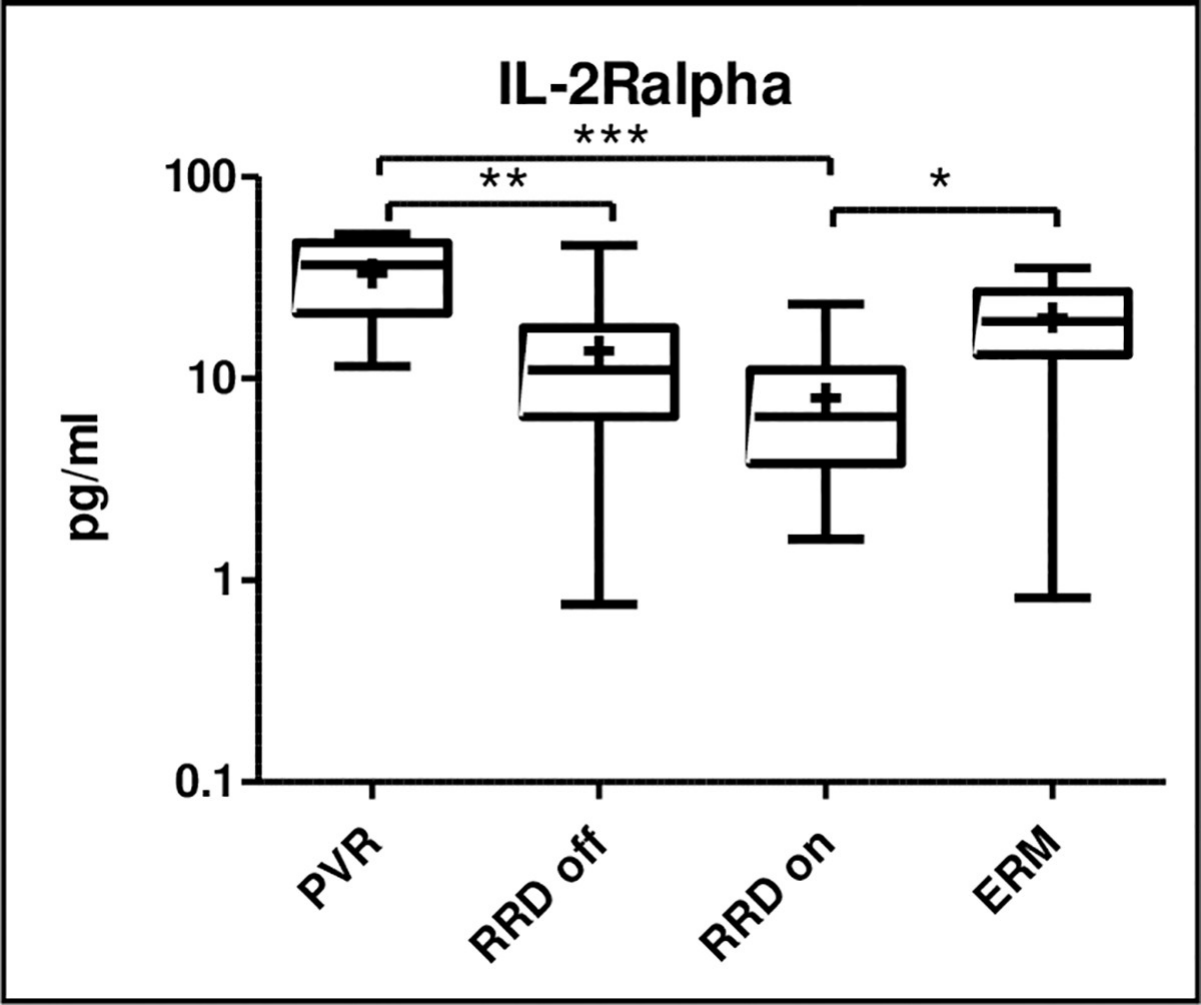
IL-2 Ralpha upregulated in PVR compared to macula off and on RRD, and the concentration is significantly lower in macula on RRD compared to ERM. Median and mean (cross) concentrations of IL-2Ralpha in eyes with PVR, macula off RRD, macula on RRD, and ERM. Statistically significant differences between the groups are marked by an asterisk. * p<0.05; ** p<0.01; *** p<0.001.

We found that the concentrations of three molecules were significantly lower in macula on RRD compared to ERM: IL-1alpha (p = 0.0202), IL-12(p40) (p = 0.0174), IL2-Ralpha (p<0.05). **(Figs 5 and 6)** The level of IL2-Ralpha was significantly higher in PVR compared to macula off and macula on RRD (p<0.0001) as well **(Fig 6)**.

## Discussion

We performed a prospective clinical study in 42 patients with RRD and 16 age-matched controls with ERM to evaluate the concentrations of vitreous cytokines, chemokines and growth factors and to investigate if there is a difference in the cytokine profile of macula off, macula on RRD and PVR. Our study results demonstrated that the vitreous of eyes with macula on RRD contains a substantially lower concentration of half of the analysed molecules. We are the first to report that there is a difference in the cytokine pattern of the vitreous of patients with macula off and macula on RRD. In macula on RRD, the concentrations of 14 molecules were significantly lower compared to PVR. Significant differences were found between macula on and macula off RRD in the concentrations of G-CSF and SCF.

SCF is a potent synergistic growth factor in haematopoiesis and results in augmentation of the proliferation, differentiation, and survival of haematopoietic cells. [16] [17] SCF synergy with G-CSF has important biological and clinical significance. Duarte et al. investigated the signalling pathways SCF promotes G-CSF. Cell cycle analysis revealed that increased proliferative state induced by SCF and G-CSF cotreatment was associated with the direct effect of these cytokines on cell cycle distribution. [18] The inflammatory character and synergistic effect on other chemokines of these molecules might have an impact on the physiology of retinal cells that contributes to impaired visual acuity in macula off RRD despite anatomically successful surgery.

We found that the concentrations of eight molecules (CTACK, eotaxin, G-CSF, MIG, IP-10, SCF, SCGF-beta, SDF-1alpha) were significantly higher in PVR compared to macula on RRD and ERM. These chemokines have a key role in the recruitment and function of T-lymphocytes, [19] and there are complex connections between them. From these eight chemokines, SCGF-beta reached the highest level from all the measured molecules. SCGF-beta has a burst-promoting activity and a granulocyte-macrophage (GM) colony-promoting activity on erythroid and GM progenitor cells [20] and acts synergistically with other cytokines, including G-CSF, GM-CSF and has a connection with CTACK, SCF, and IL-16 according to string database. The concentrations of four out of eight molecules were higher than 100 pg/ml: G-CSF, IP-10, MIG, SDF-1alpha. IP-10 and MIG bind to the same receptor (CXCR3). [21, 22] The CXCR3 chemokine receptor regulates the migration of Th1 lymphocytes and responds to three ligands: MIG (CXCL-9), IP-10 (CXCL-10), and I-TAC (CXCL11). [23] Chemokines play a role in wound healing. Early wound healing includes hemostasis, inflammation, and proliferation. Late wound healing is the remodelling stage. IP-10 and I-TAC play a role in the proliferation and remodelling stage. IL-8 (CXCL-8) plays a role in inflammation, MCP-1 (CCL-2) participates mainly in inflammation and proliferative phase of the early wound healing. IFN-gamma plays a role in angiogenesis. SDF-1alpha (CXCL-12) is present in all early phases of wound healing, including the proliferation phase. [24] Cytokines that are mainly present in the early phase were upregulated in all of the retinal detachment groups, but IP-10 that participates in the proliferative and remodelling phase was upregulated only in the macula off RRD and PVR group. Our findings indicate that in the pathophysiology of PVR, those chemokines have a key role that participates in wound healing, especially in the late phase.

The concentrations of HGF, IFN-gamma, IL-6, IL-16, MIF, MCP-1 were significantly higher in all groups of retinal detachment compared to controls. The level of IL-8 was significantly higher in macula off RRD and PVR compared to ERM. HGF, MIF, and MCP-1 had higher concentrations than 1 ng/ml in the vitreous of macula on, macula off RRD, and PVR.

HGF is one of the cytokines constitutively produced by human bone marrow (BM) stromal cells and indirectly promotes haematopoiesis. [25] Matsuda-Hashii et al. studied the effect of HGF on stromal cells. They revealed that HGF is an autocrine regulator, which is able to maintain the hematopoietic microenvironment through stimulating proliferation and adhesion to the extracellular matrix and promoting hematopoiesis through inducing constitutive production of IL-11, SDF-1alpha, and SCF. [26] Lashkari et al. investigated the role of HGF in the formation of PVR in human donor eyes. They concluded that HGF is a potent chemoattractant for cultured human RPE cells, HGF and HGF receptor might play a role in the normal function of RPE cells and RPE-related diseases such as PVR. [27] Briggs et al. searched the presence of HGF in PVR membranes, in the vitreous and the subretinal fluid of eyes with PVR. They found that RPE cells respond by shape change and cell migration to HGF. [28]

Previous studies have explored molecular alterations in RRD and PVR. Pollreisz et al. explored cytokines and chemokines that were significantly upregulated in the vitreous of RRD eyes compared with ERM, including IL-6, IL-8, MCP-1, IP-10. [1] Takahashi et al. characterized the expression profiles of 27 cytokines in the vitreous of patients with RRD compared to proliferative diabetic retinopathy (PDR), retinal vein occlusion, MH, and ERM. The levels of IL-6, IL-8, MCP-1, IP-10, MIP-1beta were significantly higher in RRD compared to the control MH group as in our study. [14] Abu El-Asrar et al. measured the levels of ten chemokines with ELISA in the vitreous from eyes undergoing pars plana vitrectomy for the treatment of RRD, PVR, and PDR and they concluded that MCP-1, IP-10, and SDF-1 might participate in the pathogenesis of PVR and PDR. [29]

Wladis et al. documented ten molecules that were statistically significantly different in PVR compared to primary RRD and ERM. The levels of IP-10, SCGF, SCF, G-CSF were higher in PVR compared to RRD and ERM in parallel with our study. [30] Roybal et al. revealed that in late PVR vitreous, cytokines driving mainly monocyte responses and stem-cell recruitment (SDF-1). [31]

Garweg et al. documented that the levels of 39 of 43 cytokines in the vitreous and 23 of 43 cytokines in the aqueous humour were significantly higher in eyes with RRD than in those with MH and they could not find relevant differences in the cytokine profiles of phakic and pseudophakic eyes. [32] Zandi et al. evaluated the same 43 cytokines in RRD, moderate, and advanced PVR compared to MH. They revealed that eyes with PVR C2-D showed higher levels of CCL27 (CTACK), CXCL12 (SDF-1), CXCL10 (IP-10), CXCL9 (MIG), CXCL6, IL-4, IL-16, CCL8 (MCP-2), CCL22, CCL15 (MIP-1delta), CCL19 (MIP-3beta), CCL23 and compared to controls. Interestingly, no difference in cytokine levels was detected between C1 and C2-D PVR. [15] They concluded that CCL19 may represent a potential biomarker for early PVR progression. [33]

In our study, we could not detect a significant difference of VEGF between the groups, but Rasier et al. demonstrated increased levels of IL-8 and VEGF in vitreous samples from eyes with RRD compared to MH and ERM. [34] Ricker et al. documented among six molecules the concentration of VEGF in the subretinal fluid was significantly higher in PVR compared to RRD.[35] Josifovska et al. studied 105 inflammatory cytokines in the subretinal fluid of 12 patients with RRD. They found that 37 of the studied cytokines were significantly higher in the subretinal fluid of RRD patients compared to the vitreous of non-RRD patients. [36]

Our study has some limitations, such as the complexity and a high number of cytokines that need further investigations to detect their relationships more exactly. Retinal detachments present with variable clinical features, which might contribute to the multiplex variations of cytokines in the fluids.

Given the corresponding results in the levels of cytokines in RRD and PVR in the different studies, they may represent novel therapeutic targets in the management of these diseases. According to our analysis and previous studies HGF, IFN-gamma, IL-6, IL-8, MCP-1, MIF, IP-10 may serve as biomarkers for RRD. CTACK, G-CSF, MIG, IP-10, SCF, SCGF-beta, and SDF-1alpha may participate in the pathogenesis of PVR and represent potential biomarkers for PVR. Upregulation of SCF and G-CSF in macula off RRD compared to macula on RRD may reveal molecular pathways that participate in the poorer prognosis of macula off RRD despite anatomically successful surgery.

## Supporting information

S1 Data (PDF)
